# Supplementary material for: Network Models Predict That Pyramidal Neuron Hyperexcitability and Synapse Loss in the dlPFC Lead to Age-Related Spatial Working Memory Impairment in Rhesus Monkeys
Source: Front Comput Neurosci. 2020 Jan 17;13:89. doi: 10.3389/fncom.2019.00089 (PMC6979278; doi:10.3389/fncom.2019.00089)
Supplement: Supplementary file 1 [file Data_Sheet_1.docx]

**Supplementary Material**

Ibañez S, Luebke JI, Chang W, Draguljić D and Weaver CM (2019). Network Models Predict That Pyramidal Neuron Hyperexcitability and Synapse Loss in the dlPFC Lead to Age-Related Spatial Working Memory Impairment in Rhesus Monkeys. *Front. Comput. Neurosci*. 13:89. doi: 10.3389/fncom.2019.00089

1. **DRT MODEL SIMULATIONS WITH A LESS EXCITABLE *F*-I CURVE**

We compared LHS results for an alternative, continuous activation function that satisfies the physiological requirements (see Brunel 2003) but is less excitable:

$f_{g}\left( I \right)=\left\{ \begin{matrix} 0 & I\leq0 \\ \nu_{c}\left( \frac{I}{I_{c}} \right)^{p} & 0<I<I_{c} \\ p{\cdot\nu}_{c}\left( \frac{I}{I_{c}}-\frac{p^{p}-1}{p^{p}} \right)^{1/p} & I_{c}\leq I \end{matrix} \right.$ .

This function generalizes the suprathreshold regime (when I_c_ $\leq$I), still with a concave shape and

$$p{\cdot\nu}_{c}\left( \frac{I}{I_{c}}-\frac{p^{p}-1}{p^{p}} \right)^{1/p}=\nu_{c}$$

when I = I_c_, but given by a root function of general order instead of a square root.

The DRT model simulations were repeated using $f_{g}\left( I \right)$ for the LHS regime in Table 2 in the main document (Supp. Fig. 1). We found good fits for the empirical data of the young, middle-aged and aged groups of subjects for ν_ce_ = 2.63 Hz, I_ce_ = 122 pA, and p_e_ = 4.9, 6.14, and 7.11, respectively (Supp. Fig. 1a). Therefore, with this activation function, varying p_e_ was sufficient to fit the empirical *f*-I curves of the three age groups. Setting ν_ci_ = 2.63 Hz, I_ci_ = 118 pA, and p_i_ = 50 for the network inhibitory neurons, we repeated the DRT simulations at each of the 4200 points in the LHS for p_e_ = 3, 4, 5, 6, 7, and 8. As when ν_ce_ increased in the classical (but more excitable) *f*-I curve in the main text, the number of points maintaining TPA-S decreased as p_e_ increased. Overall, points sampled in the regime became more excitable: Supplemental Figure 1d shows fewer under-excited and more over-excited cases as p_e_ increased (blue vs. red dots). Most synaptic weight trends reported in the main text were also maintained here, suggesting that our results are robust to variations in the f-I curve formulation.

**Supplementary figure 1: DRT model output with** $\boldsymbol{f}_{\boldsymbol{g}}\left( \boldsymbol{I} \right)$ **varied across the parameter space.** a) $f_{g}\left( I \right)$ fit (solid lines) to empirical AP firing rates of pyramidal neurons of young, middle-aged and aged subjects, averaged for each age group (black, blue, and red respectively, shown as mean $\pm$ SEM at each injection level). b) Number of points in each LHS maintaining TPA-S as p_e_ increased. c) Mean synaptic weights (G_EEa_, G_EEn_, G_IE_, G_EIa_, G_EIn_, and G_II_) for all points maintaining TPA-S as p_e_ increased. G_EEa_, G_EEn_, and G_IE_ values shown as purple, pink, and red open circles respectively; G_EIa_, G_EIn_, and G_II_ values shown as dark green, light green and open triangles respectively. S.E.M. bars lie beneath the symbols. d) DRT model output for 4200 points of the parameter space LHS, shown in 3D projections across the subspace of the excitatory (G_EEa_ and G_EEn_) and the inhibitory (G_IE_) synaptic weights of pyramidal neurons as their excitability increased (p_e_ = 4, 5, 6, and 7). Blue, green, yellow, and red dots represent under-excited networks, networks maintaining TPA-S, and partially and completely over-excited networks respectively.

1. **DRT YOUNG MODEL COHORT SIMULATIONS: ADDING THE STIMULUS CURRENT REDUCTION**

**Supplementary figure 2: Perturbing the DRT young model cohort, with reduction of input stimulus.** As in Fig. 4, the young cohort was defined as networks from the LHS maintaining TPA-S when ν_ce_ = 5 Hz (dark gray bar in Fig. 3d, and bar for young simulations in all panels in this figure). For middle-aged and aged simulations. we reduced the stimulus scaling factor, I_st_, by 10% and 30% respectively, in addition to: (a) Applied the synapse condition to young cohort, holding the excitability parameters constant. (b) Applied both observed data conditions simultaneously (excitability and synapse conditions). (c) and (d): Analogous perturbations as in (a) and (b), after adding synaptic facilitation to each network.
